# Polatuzumab Vedotin in a Patient with Refractory Burkitt Lymphoma, a Case Report

**DOI:** 10.2147/OTT.S394193

**Published:** 2023-02-21

**Authors:** Meshaal Alanzi, Mohammad Abu-Tineh, Lajos Szabados, M Z Sharaf Eldean, Sali Alatasi, Ruba Y Taha, Sarah A Elkourashy

**Affiliations:** 1 Department of Internal Medicine, Hamad Medical Corporation, Doha, Qatar; 2 Department of Medical Oncology/Hematology, National Center for Cancer Care and Research, Doha, Qatar; 3 Department of Nuclear Medicine, National Center for Cancer Care and Research, Hamad Medical Corporation, Doha, Qatar; 4 Department of Pathology, Hamad Medical Corporation, Doha, Qatar; 5 Weill Cornell Medicine University, Doha, Qatar

**Keywords:** Burkitt’s lymphoma, polatuzumab, Lymphoma, anti CD79b, resistant lymphoma

## Abstract

Although Burkitt lymphoma is considered a curable disease due to the progress made in choosing the most effective first-line therapy, relapsed or refractory Burkitt lymphoma (BL) has a very poor outcome. There is a lack of data supporting the treatment regimens. We report a 48-year-old male with stage II Burkitt’s lymphoma with no response to the first line of high-intensity chemotherapy. However, treatment with polatuzumab vedotin led to complete clinical remission for more than one year.

## Introduction

Burkitt lymphoma (BL) is a highly aggressive B-cell lymphoma. Over the years, many treatment strategies have been developed, leading to good outcomes. Although most patients are cured with intensive combination chemotherapy, optimal therapy for resistant/relapsed BL has not been defined, given the paucity of randomized trials. The prognosis of resistant/relapsed BL is very poor, with a 1-year overall survival rate not exceeding 11%, median overall survival of 2.8 months and a relapse-free survival rate of 18% only.^1^ Besides, all patients with refractory HIV-related BL have died in a 4-year follow-up in Brazil.^2^ Treatment options for refractory or relapsed disease remain an unmet need. Polatuzumab vedotin could be an exciting new therapeutic option. Polatuzumab vedotin is a new antibody-drug targeting CD79b that was approved to be used in adults with relapsed or refractory diffuse large B-cell lymphoma.^3^ There are no records of using Polatuzumab for the treatment of BL. We are reporting a case of a 48-year-old male with Stage II BL with no response to two lines of chemoimmunotherapy and responded only after the administration of polatuzumab vedotin-based chemotherapy, with progression-free survival PFS of more than one year.

## Case Presentation

A 48-year-old Filipino male with no past medical history presented in November/2019 with a large non-painful right-sided neck mass that had progressed over six months. He did not complain of fever, night sweats, or weight loss. Vital signs were within normal limits. The physical examination was significant for a mass located on the right side of the neck, around 20 cm in size, non-tender, and hard in consistency, with no redness or skin changes. Laboratory investigation reports did not show abnormalities with normal blood cell counts and kidney and liver functions. A computed tomography (CT) scan showed a right neck heterogeneous mass lesion extending from the parotid region superiorly down to the supraclavicular region inferiorly, measuring 20 cm in maximum diameter.

**Figure 1:**
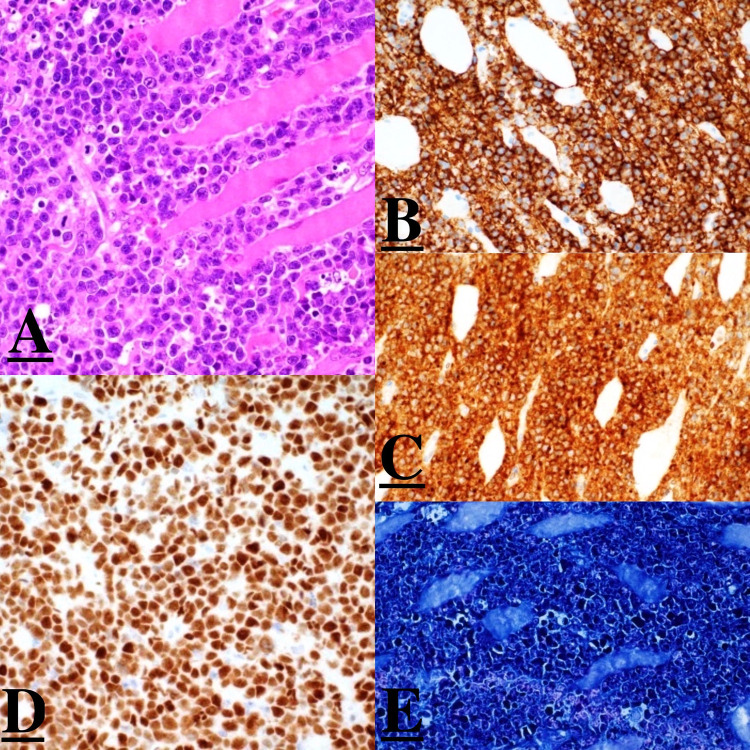
Histopathology.

**Notes**: (**A**): Skeletal muscle infiltrated by intermediate lymphocyte with large nuclei dispersed chromatin and prominent red nucleoli. Numerous mitotic figures, tangible body macrophages, and apoptotic bodies are present. Areas of necrosis are also seen. (**B**): CD20 immunohistochemical stain is positive, confirming the B-cell nature of the neoplastic cells. (**C**): The tumor cells are positive for CD10 by immunohistochemical stain. (**D**): The tumor cells are positive for MYC by immunohistochemical stain. (**E**): In situ hybridization for EBER is positive in 100% of cells. By immunohistochemical stains, the neoplastic lymphocytes are of B phenotype, positive for CD20 (**B**), CD10 (**C**), MYC (**D**), and in situ hybridization for EBER (**E**). The proliferation marker Ki-67 is positive in 100% of cells. The neoplastic cells are negative for TdT, BCL2, MUM-1, CD30, CD138, ALK-1, Cyclin-D1, and HHV-8. Fluorescent in situ hybridization (FISH) test showed the tumor cells are positive for MYC-IGH rearrangement; however, they are negative for BCL2 and BCL6 rearrangements.

An excisional biopsy was taken, and it confirmed the diagnosis as Burkitt lymphoma. The Fluorescence In Situ Hybridization (FISH) test of the biopsy came positive for MYC-IGH rearrangement and negative for BCL6 & IGH-BCL2 rearrangement. Using the in-situ hybridization (ISH), the Epstein-Barr encoding region (EBER) came positive (Figure 1). Bone marrow aspiration and biopsy revealed no evidence of bone marrow involvement. Lumbar puncture and CSF analysis were negative for malignant cells.

**Figure 2:**
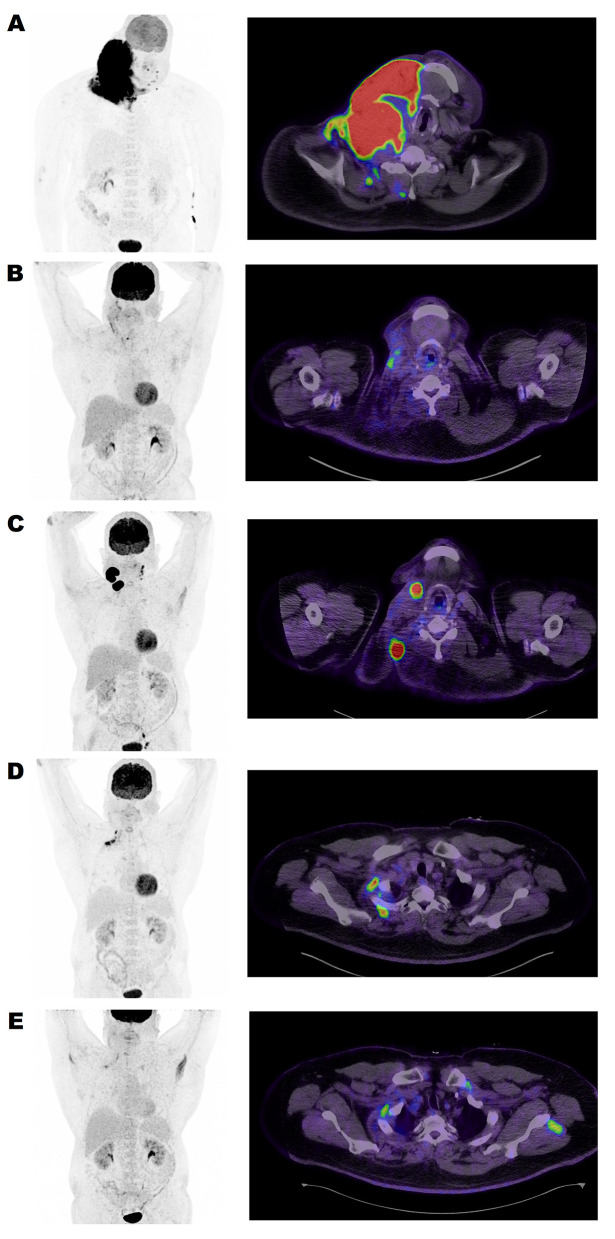
FDG PET/CT maximum intensity projection (left column) and fused PET and CT transaxial images (right column) from the lower neck region.

**Notes: (**A) (PET/CT on diagnosis) Large bulky infiltrative right lower neck mass with intense FDG uptake at primary presentation. Metabolic tumor volume (MTV) 894 cm3 measured at 40% of SUVmax. (**B**): (PET/CT post the first-line chemotherapy Magrath regimen) revealed Deauville 4-level residual uptakes. MTV 22 cm^3^. (**C**): (PET/CT post the second-line chemotherapy) A follow-up PET, FDG avid relapse was seen at the primarily involved location and on the left neck. MTV 57.6 cm^3^. (**D**): (PET/CT post three cycles of PBR protocol) Intense uptake along the right upper ribs still represents lymphoma involvement. MTV 2.8 cm3. (**E**) (PET/CT post six cycles of PBR protocol) Still Deauville 4-level residual uptake at the same location as previously. MTV 2.2 cm3 Benign muscular uptakes are also seen on both shoulders.

The FDG PET scan showed intense uptake (SUVmax 29.6) in an irregular infiltrative mass on the right side of the neck with a 20 cm maximum diameter (Metabolic tumor volume (MTV) 894 cm^3^) with no evidence of increased uptake in other regions (Figure 2A). The final diagnosis was Burkitt lymphoma stage II, bulky disease, and the multidisciplinary team (MDT) decided to start the patient on a Magrath regimen;^4^ Rituximab plus CODOX-M (cyclophosphamide, vincristine, doxorubicin, and high-dose methotrexate) with IVAC (ifosfamide, cytarabine, etoposide, and intrathecal methotrexate). From December/2019 to February/2020, he received four cycles with intrathecal methotrexate and cytarabine. Post-chemotherapy FDG PET scan showed significantly decreasing extent and uptake of right cervical lymphomatous mass overall, considered as a partial response (now with 5×4.5 maximum diameters and >50% SUVmax decreased uptake) with still residual uptake in right supraclavicular lymph nodes (SUVmax of 8.4) (Figure 2B). A repeated biopsy of the residual lesion showed abundant necrosis with histiocytes and rare multinucleated giant cells, with no viable residual Burkitt lymphoma in the examined sections. Then the patient was offered radiotherapy as a kind of consolidation. In May/2020, he received 50 Gy in 25 fractions [Intensity-modulated radiation therapy (Volumetric modulated arc therapy “Rapid Arc”)]. Three months later, the repeated FDG PET scan showed persistent hypermetabolic uptake in the right cervical mass with a small decrease in size.

As the MTV was reduced after chemotherapy to 22 cm^3^ (>98% decrease) and the patient was symptom-free and doing well, we decided to keep the patient on surveillance by CT scan every six months. However, in November/2020, the patient presented with the regrowth of 2 lumps in the same site as the previous tumor. Restaging with FDG PET scan showed progression/relapse of the disease demonstrated in two large right neck masses (anterior 3.7 cm and posterior 5.3 cm axial diameters) showing intense FDG uptake (SUVmax 32.9) with left palatine tonsil involvement, left neck level II–III lymph nodal, and left iliac and inguinal lymph nodal involvement.

A mass biopsy was done, which confirmed Burkitt lymphoma (neoplastic B-cells positive for CD-20, CD-10, MYC), and a repeated bone marrow aspiration and biopsy did not reveal any evidence of bone marrow involvement. The MDT recommended starting high-intensity therapy with a platinum-based regimen followed by an autologous stem cell transplant. An allogenic bone marrow transplant was not feasible in his case as he had only one sibling who was inaccessible. From December 2020 to January 2021, he received three cycles of the R-DHAP regimen (rituximab, dexamethasone, high-dose cytarabine, and cisplatin).^5^ Stem cell harvest was attempted in January/2021; however, it failed after two doses of plerixafor.

A follow-up FDG PET scan showed newly developed hypermetabolic lesions in the right neck and upper thorax area, suspicious of lymphoma manifestation (Figure 2C). The MDT discussed the case as a transplant-ineligible refractory Burkitt lymphoma who did not respond to two lines of chemoimmunotherapy. The final recommendation was to manage the case with palliative chemotherapy and supportive care. In a non-transplant candidate with excellent performance with {the Eastern Cooperative Oncology Group performance scale of 0–1}, we suggested initiating a polatuzumab-based regimen as a case study to control the disease and prolong survival. The patient agreed and consented to such off-label, non-formulary treatment. He was started on the polatuzumab vedotin (1.8 mg/kg) plus Bendamustine (90 mg/m^2^) and Rituximab (375 mg/m^2^) (PBR) protocol. After three cycles, an FDG PET scan showed uptake in the previously seen right lower neck and thoracic inlet lesions; however, MTV reduced from 57.6 cm^3^ to 2.8 cm^3^(Figure 2D). The patient tolerated the treatment well, maintaining good performance apart from prolonged neutropenia secondary to Bendamustine.

Then the FDG PET scan was repeated after completing six cycles of PBR protocol and showed further decreasing uptake in the previous lesions with no new ones (MTV 2.2 cm^3^) (Figure 2E). Then, two more doses of Polatuzumab and Rituximab were additionally administered. A follow-up CT scan with contrast, three months after completion of treatment, showed a decrease in the size of the right-sided cervical masses and lymph nodes. No further complications were encountered, and blood counts started to improve gradually. He was kept on a prophylactic dose of sulfamethoxazole-trimethoprim with oral Valacyclovir. He resumed his full-time job and regular daily activities. After more than one year since the last treatment, there is no clinical evidence of any recurrence, and the patient enjoys his life with no symptoms.

## Discussion

Burkitt lymphoma is a highly aggressive B-cell non-Hodgkin’s lymphoma first described by Denis Burkitt in African children in 1958.^6^ It is characterized by the translocation of the MYC proto-oncogene, loculated on chromosome 8q24.^7^ BL is divided into three epidemiological and clinical subtypes: endemic (African), sporadic, and immunodeficiency associated.

Multi-agent Chemotherapy is the main method of therapy used to manage BL. Over the years, different chemotherapy protocols were studied, but none was considered a standard of care.^8^,^9^

The National Comprehensive Cancer Network (NCCN) recently updated guidelines recommending treating patients based on risk stratification and age.^10^ After assessing the patient’s risk, they recommended using one of the first-line chemotherapy protocols, which included CODOX-M/IVAC with or without rituximab, CODOX-M (combination of cyclophosphamide, vincristine, doxorubicin, prednisone, and systemic high-dose methotrexate [MTX]) with IVAC (ifosfamide, mesna, etoposide, cytarabine, and IT MTX). This regimen results in two- and five-year progression-free survival of 78% and 75%, respectively, with 2- and 5-year overall survival of 81% and 77%, respectively.^11^ The other option is HyperCVAD (cyclophosphamide, vincristine, doxorubicin, and dexamethasone) alternating with high-dose methotrexate, cytarabine, and rituximab (regimen includes intrathecal therapy). Lastly, the dose-adjusted EPOCH protocol (which includes prednisone, etoposide, vincristine, cyclophosphamide, and doxorubicin) plus rituximab was evaluated in a prospective study where the results showed event-free survival and overall survival of 84.5% and 87.0%, respectively, with event-free survival of 100% in low-risk and 82.1% in high-risk patients.^12^

On the other hand, there are limited data on managing patients with relapsed or resistant BL. In the NCCN guidelines, using second-line chemotherapy that was not used before is suggested, referring these patients to clinical trials or best supportive care based on the time of relapse.^10^ Options for second-line chemotherapy include dose-adjusted EPOCH, RICE (rituximab, ifosfamide, carboplatin, etoposide), intrathecal methotrexate if not given previously; RIVAC (rituximab, ifosfamide, cytarabine, etoposide); intrathecal methotrexate if not given previously; High-dose therapy with autologous stem cell rescue plus/minus involved-site radiation therapy (ISRT) is considered for a patient who has relapsed after six months of remission and had a complete response to second-line chemotherapy.

Polatuzumab vedotin is an antibody-drug conjugate (ADC) consisting of a combination of an anti-CD79b monoclonal antibody and the cytotoxic drug monomethyl auristatin E (MMAE), a potent microtubule inhibitor.^13^,^14^ CD79b is a subunit of a heterodimer transmembrane component of the B-cell antigen receptor involved in cell signaling. It is predominantly expressed on the surface of mature B-cell lymphomas.^15^,^16^ Polatuzumab vedotin binds to the B cell receptor through CD79b; the cytotoxic drug is released inside the B cell.^17^ Polatuzumab vedotin was used in combination with bendamustine and rituximab (PBR protocol) for the treatment of patients with transplantation-ineligible relapsed or refractory diffuse large B-cell lymphoma (DLBCL).^18^ This combination resulted in a 58% reduction in the risk of death compared with bendamustine and rituximab alone.^19^ As a result, the Food and Drug Administration (FDA) approved the use of this combination in the treatment of relapsed or refractory (DLBCL).^3^,^16^

To our knowledge, polatuzumab vedotin was never used to manage patients with Burkitt lymphoma. However, a pre-clinical trial compared the use of polatuzumab vedotin alone or in combination with rituximab, Obinutuzumab, and ex-vivo expanded allogeneic natural killer cells showed that adding polatuzumab vedotin alone or in combination with Obinutuzumab induces cytotoxicity and possibly improves survival against BL and rituximab-resistant BL.^20^

Polatuzumab vedotin does not bind CD79b in animals, only humans. As a result, it is not feasible to assess its efficacy in animals. However, in literature, one trial was conducted on a cynomolgus monkey using a polatuzumab-similar antibody-drug conjugate revealed a dose-dependent tumor suppressive effect toward the human Burkitt lymphoma‐derived B‐cells, and they suggested the involvement of polatuzumab vedotin in clinical trials.^21^

We adopted the same protocol approved by the FDA to treat DLBCL for our middle-aged transplant-ineligible BL patient,^3^ who is fit with excellent performance status, based on the previously mentioned results in relapsed/refractory DLBCL patients. Otherwise, the other option would be palliative and supportive care. Fortunately, it resulted in a good response, and he only attained remission after such treatment. He is now surviving more than one-year post-completion of the PBR protocol, leading a normal life and performing everyday activities.

## Conclusion

The treatment choices for patients with Burkitt lymphoma refractory to first- and second-line chemotherapy are limited. We report the first use of polatuzumab vedotin in resistant/recurrent BL with an appropriate response. However, further research is required to determine its efficacy and adverse effects.

## Data Sharing Statement

Data and materials are available upon reasonable request.
